# Non-functioning oxyphilic parathyroid carcinoma: a case report

**DOI:** 10.1186/s40792-021-01201-y

**Published:** 2021-05-12

**Authors:** Masaomi Sen, Ryuta Nagaoka, Hiroko Kazusaka, Mami Matsui, Marie Saitou, Iwao Sugitani, Takashi Sakatani, Kaori Kameyama

**Affiliations:** 1 Department of Endocrine Surgery, Nippon Medical School Hospital, 1-1-5 Sendagi, Bunkyo-ku, Tokyo, 113-8603 Japan; 2 Department of Diagnostic Pathology, Nippon Medical School Hospital, 1-1-5 Sendagi, Bunkyo-Ku, Tokyo, 113-8603 Japan; 3 Department of Pathology, Showa University Northern Yokohama Hospital, 35-1 Chigasakichuo, Tsuzuki-ku, Yokohama-shi, Kanagawa, Tokyo 224-8503 Japan

**Keywords:** Non-functioning, Parathyroid carcinoma, Chromogranin A, PTH, Parafibromin

## Abstract

**Background:**

Non-functioning parathyroid carcinoma is an extremely rare malignancy among endocrine tumors. We report a case in which non-functional oxyphilic parathyroid carcinoma was diagnosed from clinical symptoms and pathological diagnosis.

**Case presentation:**

The patient was a 42-year-old man with no medical or family history of note. He had presented to a local hospital with a neck mass 2 months earlier. Medullary thyroid carcinoma was diagnosed and he was referred to our department. A 3.5-cm mass was observed in the left thyroid lobe. Laboratory data for thyroid functions, thyroglobulin, anti-thyroglobulin antibodies, anti-thyroid peroxidase antibodies, serum calcium, and parathyroid hormone (PTH) were all within normal ranges. Ultrasonography revealed a 40-mm irregular, hypoechoic mass throughout the left thyroid lobe. Follicular thyroid tumor was suspected from fine-needle aspiration cytology. Left lobectomy was performed. Pathological features revealed a thick fibrous capsule around the tumor, and a thick fibrous band was observed inside the tumor. Both capsular invasions and vascular invasions were observed. Tumor cells were eosinophilic and displayed solid growth. Immunohistochemically, tumor cells were negative for thyroid transcription factor-1, negative for thyroglobulin, negative for chromogranin A (positive for normal parathyroid tissue within the nodule), positive for PTH, and positive for parafibromin. Ki-67 labeling index was 10%. Based on these findings, non-functional oxyphilic parathyroid carcinoma was diagnosed. One and a half years postoperatively, calcium and PTH were within normal ranges, and he has shown no evidence of recurrence or metastasis.

**Conclusions:**

Non-functioning oxyphilic parathyroid carcinoma is an extremely rare malignancy, and definitive diagnosis is difficult to obtain preoperatively. Few reports have been made worldwide, and information on the long-term prognosis is scarce. Long-term surveillance by imaging is mandatory, since no indices that can be used as a marker for postoperative recurrence and metastasis have been identified.

## Background

Non-functioning parathyroid carcinoma (PC) refers to PC without clinical or laboratory findings of primary hyperparathyroidism. This entity is an extremely rare endocrine malignancy, accounting for less than 0.055% of parathyroid tumors [1, 2]. Preoperative diagnosis is very difficult. Pathological diagnosis by immunostaining is useful for diagnosing this disease. We report a case in which non-functional oxyphilic PC was diagnosed from clinical symptoms and pathological examinations.

## Case presentation

**Fig. 1:**
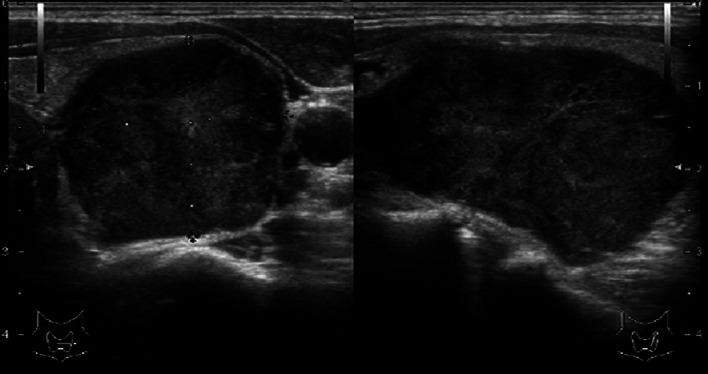
US findings in the neck. An irregularly shaped hypoechoic mass measuring 46 × 29 × 24 mm in diameter without clear halo is seen in the left lobe of the thyroid gland

**Fig. 2:**
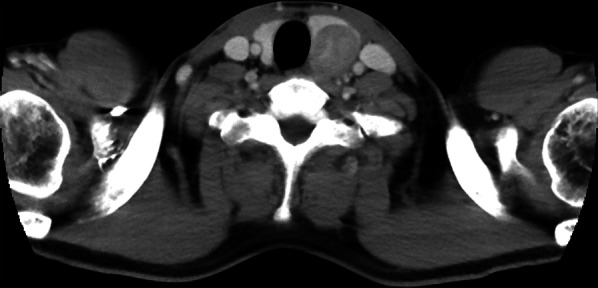
Enhanced CT of the neck. The mass lesion shows poor contrast on the posterior side of the left lobe of the thyroid. No extraglandular infiltration or cervical lymphadenopathy is observed

**Fig. 3:**
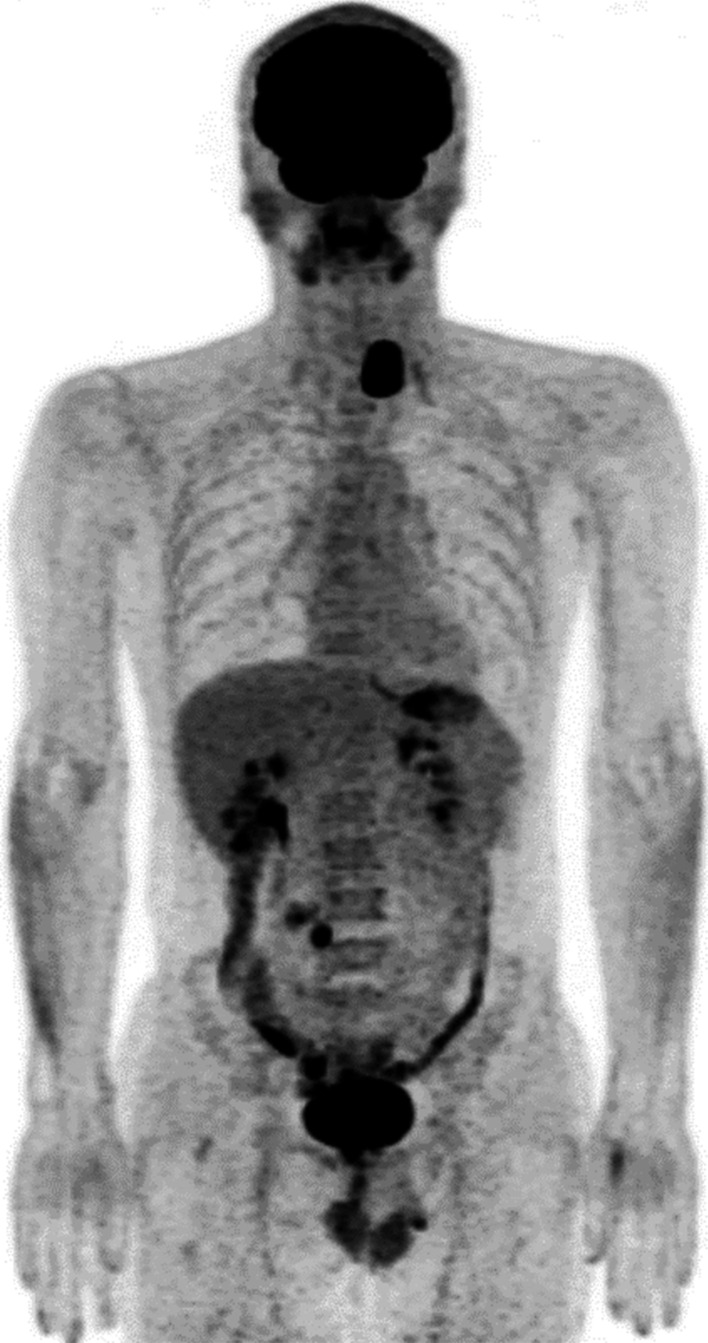
PET–CT. Abnormal accumulation is seen in the left thyroid lobe (SUVmax, 23.90), no other abnormalities

**Fig. 4:**
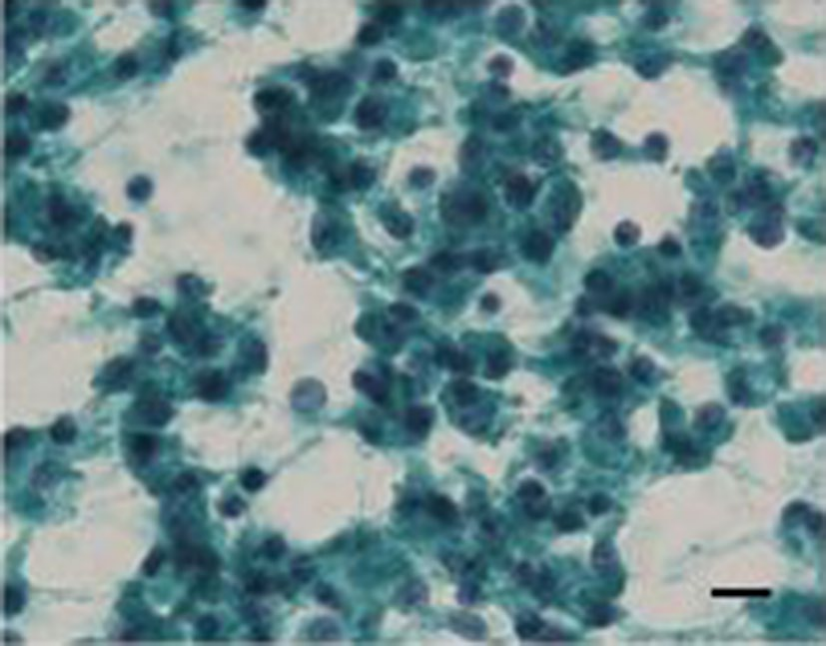
FNAC. Granular, short spindle-shaped and atypical cells with clear nucleoli (scale bar 10 μm)

**Fig. 5:**
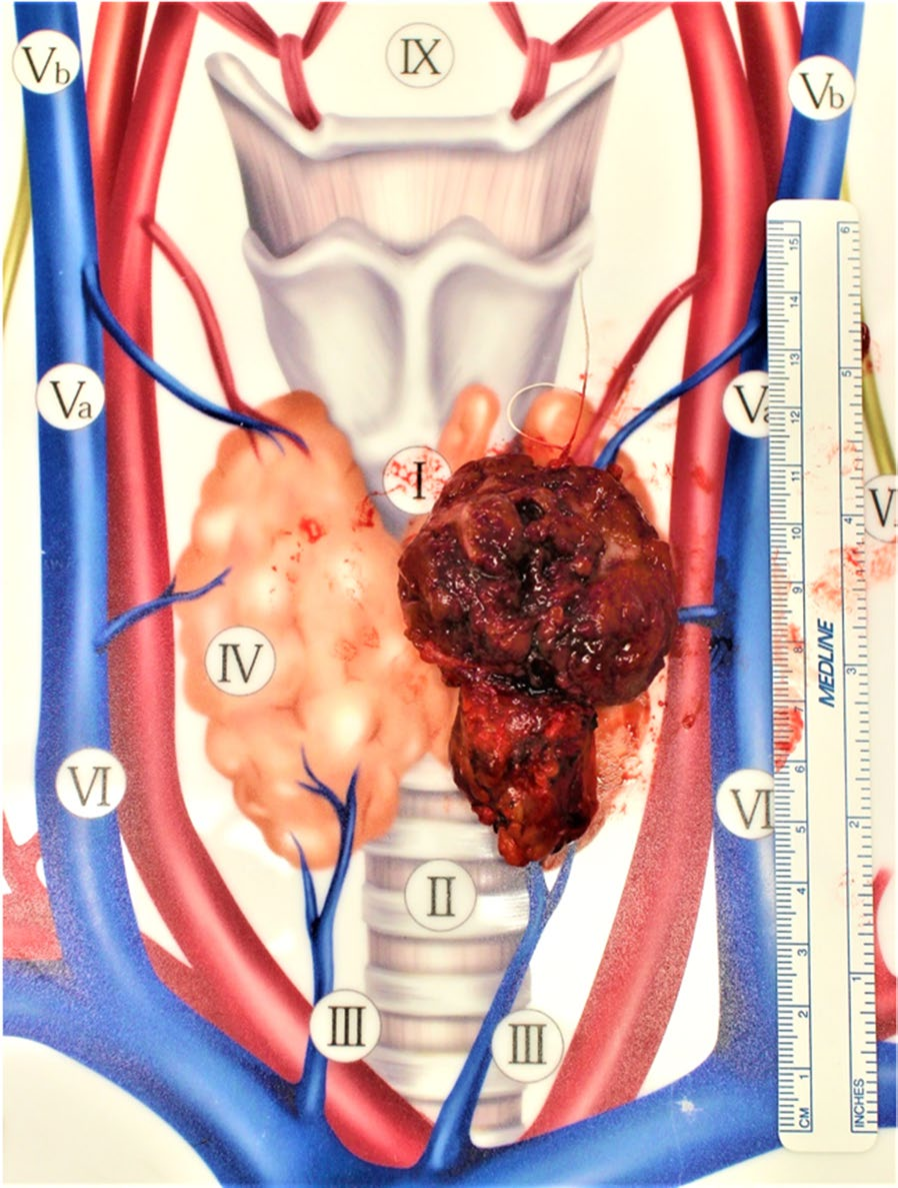
Resected specimen. An irregularly marginated, lobulated mass measuring 40 mm in diameter was found occupying the whole left lobe, and the cut surface was reddish-brown and solid

**Fig. 6:**
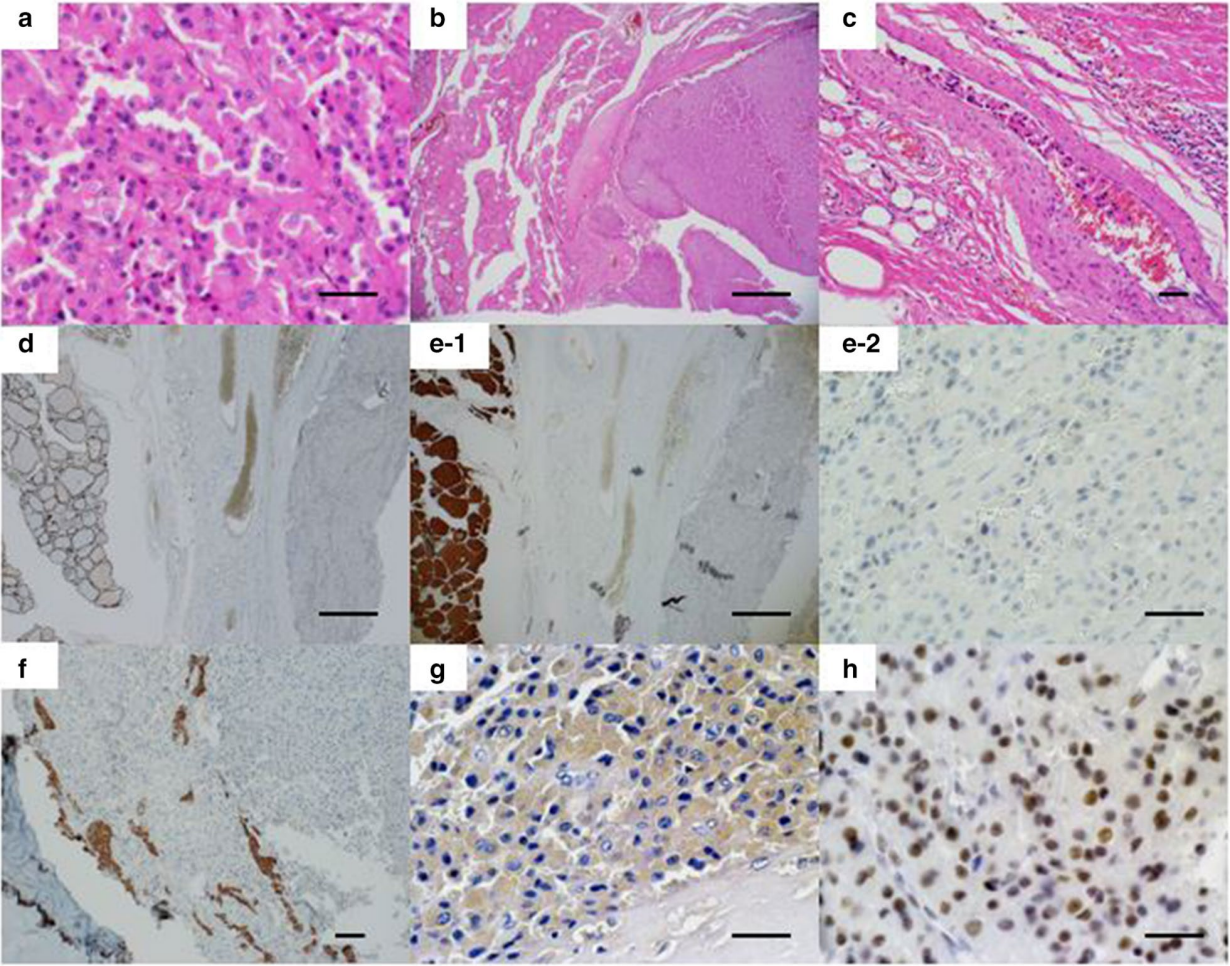
Histopathological findings. **a** Tumor cells are eosinophilic and show solid growth (scale bar 50 μm). **b** Capsular invasion (scale bar 500 μm). **c** Vascular invasion (scale bar 50 μm). **d** Negative staining for TTF-1 (scale bar 500 μm). **e** Negative staining for thyroglobulin 1: (scale bar 500 μm) 2: (scale bar 50 μm). **f** Negative staining for chromogranin A (scale bar 100 μm). **g** Positive staining for PTH (scale bar 50 μm). **h** Positive staining for parafibromin (scale bar 50 μm)

The patient was a 42-year-old man with no contributory medical or family histories. He had become aware of a neck mass 2 months earlier and visited a local hospital. Ultrasonography (US) and computed tomography (CT) showed a mass lesion in the left lobe of the thyroid gland. Fine-needle aspiration cytology (FNAC) suggested medullary thyroid carcinoma (MTC), so the patient was referred to our department. Physical examination revealed a 3.5-cm elastic hard, mobile mass in the left thyroid lobe. No other significant findings were observed. Laboratory data demonstrated: thyrotropin (TSH), 1.78 µIU/mL (normal 0.50–5.00 µIU/mL); free thyroxine (FT4), 1.38 ng/dL (normal 0.90–1.70 ng/dL), free triiodothyronine (FT3), 3.50 pg/mL (normal 2.3–4.0 pg/dL); thyroglobulin (Tg), 10.60 ng/mL (normal, < 33.7 ng/mL); anti-thyroid peroxidase antibodies (TPOAb), < 9.0 IU/mL (normal < 16.0 IU/mL); anti-thyroglobulin antibodies (TgAb), < 10.0 IU/mL (normal < 28.0 IU/mL); alkaline phosphatase, 197 U/L (normal 106–322 U/L); serum calcium, 9.7 mg/dL (normal 8.8–10.1 mg/dL); intact parathyroid hormone (PTH), 63.0 pg/mL (normal 10–65 pg/mL); carcinoembryonic antigen (CEA), 1.7 ng/mL (normal ≤ 5 ng/mL); and calcitonin, 2.54 pg/mL (normal ≤ 5.15 pg/mL). US showed a 46-mm irregularly shaped hypoechoic mass without clear halo in the left lobe of the thyroid gland (Fig. 1). Contrast-enhanced CT revealed a mass lesion with poor contrast on the posterior side of the thyroid, with no extraglandular infiltration or cervical lymphadenopathy (Fig. 2). Positron emission tomography (PET)–CT indicated abnormal accumulation in the left thyroid lobe and no other abnormalities (Fig. 3). We reviewed the FNAC specimen in the Department of Pathology of our hospital, and follicular thyroid tumor was suspected based on the granular, short spindle-shaped and atypical cells with clear nucleoli (Fig. 4). We then performed left lobectomy. Adhesions were identified between the tumor and surrounding connective tissues. An irregularly marginated, lobulated mass measuring 40 mm in diameter was found occupying the whole left lobe, and the cut surface was reddish-brown and solid (Fig. 5). Pathological findings revealed a thick, fibrous capsule around the tumor, and a thick fibrous band was observed inside the tumor. Both capsular invasions and vascular invasions were observed. Tumor cells were eosinophilic and exhibited solid growth, with anisocytotic, round nuclei of different sizes and increased chromatin. Mitotic figures were not clear. Immunohistochemically, tumor cells were negative for thyroid transcription factor-1 (TTF-1), negative for thyroglobulin, negative for chromogranin A (positive for normal parathyroid tissue within the nodule), positive for PTH, and positive for parafibromin. Ki-67 labeling index was 10% (Fig. 6). Based on the above findings, non-functional oxyphilic PC was diagnosed. No complications such as postoperative bleeding, recurrent laryngeal nerve palsy, or hypoparathyroidism were seen, and the patient was discharged on postoperative day 4. As of one and a half years postoperatively, serum Ca and PTH levels were within normal ranges, and imaging studies showed no evidence of recurrence or metastasis.

## Discussion

Non-functioning PC is an extremely rare endocrine malignancy. Most patients with PC show functioning tumors and thus display symptoms related to hypercalcemia. In contrast, patients with non-functioning PC do not show such distinct symptoms, but have a palpable neck mass, hoarseness, dyspnea, dysphagia, and decreased range of motion in the neck [3]. Thyroid tumor was suspected because of the palpable neck mass and the lack of symptoms related to primary hyperparathyroidism. Blood test findings were within normal ranges. Neck US suggested malignancy, but non-functioning PC is difficult to include among the differential diagnoses. When parathyroid tumors, particularly PC, are suspected, FNAC is contraindicated because of the risk for tumor cell dissemination [4]. Moreover, FNAC may also fail to distinguish parathyroid tumor from a benign thyroid nodule because parathyroid and thyroid lesions display some morphological similarities in cytology [5]. In this case, FNAC was performed under a presumptive diagnosis of thyroid tumor and diagnosed suspected follicular thyroid tumor.

The histopathological criteria for factors to determine the malignancy of PC as proposed by Schantz and Castleman included: (1) fibrous trabeculae; (2) mitotic figures; (3) capsular invasion; and (4) blood vessel invasion [6]. The diagnostic criteria for PC and follicular thyroid carcinoma are actually very similar. Thyroid tissue and parathyroid tissue thus need to be differentiated by immunohistochemical examination. Yamashita et al. reported that immunostaining for PTH and chromogranin A was useful for differentiating non-functioning PC from thyroid carcinoma and thymoma [7]. However, oxyphilic cells are not stained by chromogranin A and PTH is less stainable than chief cells. In this case, tumor cells were eosinophilic on hematoxylin and eosin (HE) staining, negative for TTF-1, thyroglobulin, and chromogranin A, and positive for PTH on immunostaining. Clinical and pathological findings led to a diagnosis of non-functioning oxyphilic PC.

Parafibromin is a protein encoded by the *HRPT2* gene and is present in the parathyroid gland, and *HRPT2* gene mutation has been identified in hyperparathyroidism–jaw tumor syndrome (HPT–JT syndrome) [8]. Loss of parafibromin is seen significantly more often in PC than in other parathyroid lesions and may be useful for differential diagnosis [9, 10]. This loss was reported to be linked to unfavorable clinical outcomes and mortality [11, 12]. This case was positive for parafibromin and might show low-grade malignant potential.

Oxyphilic PC is a rare histopathological subtype. Two cases of oxyphilic PCs were first reported by Obara et al. in 1985. Both cases were functioning [13]. Lori et al. summarized the clinicopathological and immunostaining characteristics for 10 cases of functioning oxyphilic PCs in 2002. They showed similar clinical outcomes to chief cell PC [14]. This case involved oxyphilic PC, but showed no function and was very rare clinically and pathologically.

The prognosis of non-functioning PC is usually poor because of detection at advanced stages, the relative ineffectiveness of adjuvant treatment modalities, and the lack of serum markers for clinical follow-up [3]. For patients with non-functioning PC, postoperative surveillance requires periodic imaging studies including neck US, lung CT, and/or fluorodeoxyglucose PET.

## Conclusions

Non-functioning oxyphilic PC is an extremely rare malignancy, and is difficult to definitely diagnose preoperatively. Few reports have been made worldwide, and information on the long-term prognosis is scarce. Long-term surveillance by imaging is mandatory, since indices that can be used as markers for postoperative recurrence and metastasis are yet to be identified.

## Data Availability

The authors declare that all data related to this article are available in this manuscript.

## Abbreviations

PC: Parathyroid carcinoma
US: Ultrasonography
CT: Computed tomography
FNAC: Fine-needle aspiration cytology
MTC: Medullary thyroid carcinoma
TSH: Thyrotropin
FT4: Free thyroxine
FT3: Free triiodothyronine
TPOAb: Anti-thyroid peroxidase antibodies
TgAb: Anti-thyroglobulin antibodies
CEA: Carcinoembryonic antigen
PTH: Parathyroid hormone
PET: Positron emission tomography
TTF-1: Thyroid transcription factor-1
HE: Hematoxylin and eosin
HPT–JT syndrome: Hyperparathyroidism–jaw tumor syndrome
